# Development of a multiplex RT‐RPA assay for simultaneous detection of three viruses in cucurbits

**DOI:** 10.1111/mpp.13380

**Published:** 2023-07-18

**Authors:** A. Abdul Kader Jailani, Mathews L. Paret

**Affiliations:** ^1^ North Florida Research and Education Center University of Florida Quincy Florida USA; ^2^ Plant Pathology Department University of Florida Gainesville Florida USA

**Keywords:** begomovirus, crinivirus, cucurbit, isothermal, plant diagnosis, squash, watermelon

## Abstract

Begomoviruses and criniviruses, vectored by whiteflies (*Bemisia tabaci*), are important threats to crops worldwide. In recent years, the spread of cucurbit leaf crumple virus (CuLCrV), cucurbit yellow stunting disorder virus (CYSDV) and cucurbit chlorotic yellows virus (CCYV) on cucurbit crops has been reported to cause devastating crop losses in many regions of the world. In this study, a multiplex recombinase polymerase amplification (RPA) assay, an isothermal technique for rapid and simultaneous detection of DNA and RNA viruses CuLCrV, CYSDV and CCYV was developed. Highly specific and sensitive multiplex RPA primers for the coat protein region of these viruses were created and evaluated. The sensitivity of the multiplex RPA assay was examined using serially diluted plasmid containing the target regions. The results demonstrated that multiplex RPA primers have high sensitivity with a detection limit of a single copy of the viruses. The multiplex RPA primers were specific to the target as indicated by testing against other begomoviruses, potyviruses and an ilarvirus, and no nonspecific amplifications were noted. The primers simultaneously detected mixed infection of CCYV, CYSDV and CuLCrV in watermelon and squash crude extracts. This study is the first report of a multiplex RPA assay for simultaneous detection of mixed infection of DNA and RNA plant viruses.

## Graphical abstract

Detection of whitefly‐transmitted cucurbit‐infecting viruses using a rapid multiplex recombinase polymerase amplification‐based isothermal assay.

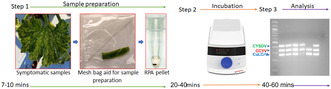

Many plants in the *Cucurbitaceae* family are high‐value crops that are grown worldwide. The family comprises more than 800 species, including gourds and cucurbits (cucumber, melon, pumpkin, squash and watermelon; Jeffrey, 2019). In the United States, Florida is the second‐largest cucurbit‐producing state and also the leading fresh market producer of cucumber, squash and watermelon (USDA‐NASS, 2021). The total value of cucurbits in the United States is $1.6 billion (USDA‐NASS, 2021). More than 70 different viruses infect cucurbits worldwide and cause different diseases (Lecoq & Desbiez, 2012, Lecoq & Katis, 2014). Viruses in the genus *Begomovirus* and *Crinivirus* are among the major threats to cucurbit production in the United States (Adkins et al., 2007, 2011). Many of the viruses in these genera are transmitted by whiteflies, creating losses in watermelon and squash cultivation. This includes cucurbit leaf crumple virus (CuLCrV), cucurbit yellow stunting disorder virus (CYSDV) and cucurbit chlorotic yellows virus (CCYV) (Adkins et al., 2013; Akad et al., 2008; Jailani et al., 2022; Polston et al., 2008). These viruses in single or mixed infection cause severe curling, crinkling and yellowing of the leaves, change in shape, uneven ripening, and reduced sweetness and quality of the fruits. Early detection by establishing a simple, fast and rapid diagnostic technique can help in monitoring viruses in new geographical areas or before the production season, thereby assisting producers in improving their decision‐making capacity, which may include the choice of cultural and chemical management approaches (Kavalappara et al., 2021).

Currently used diagnostic techniques for virus detection include PCR for CuLCrV, CYSDV and CCYV (Adkins et al., 2013; Akad et al., 2008; Hagen et al., 2008), reverse transcription (RT)‐PCR for CuLCrV, CYSDV, squash vein yellowing virus (SqVYV) and CCYV (Abrahamian et al., 2013; Hagen et al., 2008; Turechek et al., 2010), RT‐quantitative PCR (RT‐qPCR) for CYSDV and CCYV (RT‐qPCR; Abrahamian et al., 2013; Turechek et al., 2010), and ELISA for CYSDV and CCYV (Abrahamian et al., 2013). Singleplex and multiplex PCR and RT‐PCR methods can detect these viruses from as little as 1 ng of total nucleic acid. Similarly, multiplex RT‐PCR can detect as few as 10^3^ copies of viral targets from the total plant nucleic acids in a single reaction (Jailani et al., 2021). However, direct use of the plant sap as a template is not possible for initial reaction in conventional and real‐time qPCR because of inhibition of the total reaction or limited success in amplifying the viral targets. In addition, RT‐qPCR typically requires more time to perform and has high cost compared to rapid isothermal assays. This is primarily due to the additional steps involved in the process, including RNA extraction, RT, PCR amplification and analysis of results. Moreover, the above‐mentioned techniques do not detect single copies of the viruses from the plant crude sap. ELISA in general takes several hours to complete the entire procedure, cannot detect low concentration of the viruses and is known to have issues with cross‐reactivity (Livieratos et al., 1999).

New isothermal detection techniques developed for these viruses include loop‐mediated amplification (LAMP) for CuLCrV (Waliullah et al., 2020) and singleplex recombinase polymerase amplification (RPA) assays for CuLCrV (movement protein [MP]) and CYSDV (coat protein [CP]; Kalischuk et al., 2020, 2021). The LAMP and RPA assays have been shown to be highly specific and sensitive. They can work at constant temperatures and do not require a thermal cycling machine. Direct plant material can also be used as a template for the initial reaction, as purification of DNA and RNA is not required. However, the isothermal diagnostic techniques developed so far for these viruses are a singleplex detection system. This does not allow the detection of many viruses at the same time and increases the output time for results in cases of mixed infections. A multiplex detection system, if developed, could overcome these limitations by detecting multiple targets in a single‐tube reaction. Most recent studies have shown that single‐virus infection is becoming rare in cucurbits in Florida and other states (Jailani et al., 2022, Jailani, DaSilva, et al., 2023, Jailani, Iriarte, et al., 2023; Kavalappara et al., 2021; Khanal et al., 2021). Many studies have reported mixed infection of two, three or four viruses in cucurbits including CuLCrV, CYSDV, SqVYV, CCYV, papaya ringspot virus‐watermelon strain (PRSV‐W), squash mosaic virus (SqMV), watermelon mosaic virus‐2 (WMV‐2), zucchini yellow mosaic virus (ZYMV), cucumber mosaic virus (CMV), watermelon crinkle leaf associated virus 1 (WCLaV1) and cucumber green mottle mosaic virus (CGMMV) (Iriarte et al., 2023). Many PCR‐based multiplex detection techniques that have been reported for the viruses mentioned above are specific but are time‐consuming, require nucleic acid purification and cannot detect single copies of the viral genome from direct sap within 20 min. To overcome these limitations, creating a fast, simple detection assay for simultaneous detection of these viruses using multiplex RPA is a possibility.

RPA assays are broadly used for the detection of human, animal and plant pathogens, including feline herpesvirus 1, *Theileria equi*, rose rosette virus and CYSDV among others (Babu et al., 2017; Kalischuk et al., 2020; Lei et al., 2020; Wang et al., 2017). The first report of multiplex RPA assay was on simultaneous detection of three bacterial pathogens (Ma et al., 2020). Also, a study reported the development of multiplex lateral flow detection RPA assay for the detection of up to seven DNA targets in a single RPA reaction (Kersting et al., 2014; Li et al., 2019). Some studies have reported the development of duplex RPA assays to detect pathogens using plant, animal and human DNA samples (Lei et al., 2020; Xia & Chen, 2020). So far, to the best of our knowledge, RPA assays for the detection of mixed DNA and RNA targets of plant viruses have not been described.

Based on these underlying scenarios, in the current study the potential development of a simple, rapid, sensitive and specific detection system for mixed DNA and RNA viruses affecting cucurbits in a single reaction tube through a one‐step conventional multiplex RPA assay was studied. The objectives of this work were to (1) develop a method to detect whitefly‐transmitted mixed DNA and RNA viruses in a single RPA reaction tube; (2) examine the sensitivity of a single‐step multiplex RPA detection system using a serially diluted plasmid control; (3) create a strong plasmid control for validation of false‐negative amplification; (4) determine the specificity of multiplex RPA primers; and (5) develop a single‐copy virus detection system for diagnosing mixed viruses from asymptomatic plants or plants showing early stages of symptoms.

In 2021, a field survey was conducted in commercial cucurbit production fields in Florida and Georgia, and at the research fields of the University of Florida, North Florida Research and Education Center (NFREC), Quincy, FL. This study monitored the presence of whitefly‐transmitted viruses on watermelon and squash that were showing virus‐like symptoms including curling, yellowing, crumpling and vein yellowing on leaves, and fruit distortions or streaks. The symptomatic leaves and fruit samples from the plants were collected. Healthy watermelon and squash plants grown in a virus‐free environment under laboratory conditions were used as negative controls. The total plant nucleic acid was isolated from symptomatic and healthy leaf samples using the RNeasy Plant Mini Kit (Qiagen) following the manufacturers' protocol. The quality and quantity of the total plant nucleic acid extracted were examined by NanoDrop spectrophotometer (Thermo Scientific) for the RT‐PCR downstream process. The quantified total nucleic acid was further tested for the presence of mixed viruses using CuLCrV, CYSDV and CCYV CP gene‐specific primers by one‐step multiplex RT‐PCR (Jailani et al., 2021).

The multiplex reverse transcriptase‐RPA (RT‐RPA) primers were designed from the highly specific CP region with a selection of specific forward and reverse primers for CuLCrV, CYSDV and CCYV. The design of the multiplex RPA primers (forward and reverse primers) did not complement or overlap with the other two sets of forward and reverse primers. The multiplex RT‐RPA primers were created using Primerquest primer designing software (Integrated DNA Technologies). The TwistDx primer designing instructions were followed to create the RT‐RPA multiplex primers (TwistDX). CuLCrV, CYSDV and CCYV CP sequences were analysed by BioEdit software to design virus‐specific RPA primers for the unique detection of CuLCrV, CYSDV and CCYV. The specificity of the CuLCrV, CYSDV and CCYV primers was further analysed through NCBI BLASTn software (http://www.ncbi.nlm.nih.gov/blast/) as well as the BioEdit tool (Hall, 1999). The multiplex RT‐RPA primers were synthesized by Integrated DNA Technologies as per standard purification protocol. The synthesized multiplex RT‐RPA primers were tested in a singleplex one‐step RT‐PCR using OneTaq One‐step RT‐PCR Kit (NEB) as per the manufacturers' instructions, and the reaction was conducted as described in the RT‐PCR programme (Jailani et al., 2021). The RT‐PCR amplicons were cloned into a pGEM‐T Easy vector (Promega) as per the protocol and the plasmid used as the positive control in the multiplex RT‐RPA reaction. The clones were further confirmed by Sanger sequencing by universal M13 forward primers (Florida State University Sequencing Facility, Tallahassee, FL, USA) and the obtained sequence similarities were confirmed by NCBI BLASTn software.

One hundred milligrams of the leaf tissue ground into 1 mL of GEB buffer (Agdia) with a 1:10 ratio (leaf:buffer) and direct plant crude extract was used as a template in the RT‐RPA reaction. The multiplex RT‐RPA primers of CuLCrV, CYSDV and CCYV (Table 1) were further evaluated for specificity through a singleplex RPA reaction using the TwistAmp Basic DNA kit (TwistDx Ltd) and 1 μL of 50× reverse transcriptase (Superscript IV; Thermo Scientific) added for RNA virus detection. The RPA ingredients and reaction conditions were used as per the TwistDx manufacturer's instructions. The freeze‐dried pellet of the singleplex reaction from the RPA TwistAmp basic kit for DNA target detection (plasmid controls or DNA or sap for CuLCrV) or TwistAmp RT‐RNA kit for RNA target detection (direct RNA or direct plant sap) was rehydrated by adding 46.5 μL of a master mix composed of 29.5 μL of rehydration buffer, 2.1 μL of each reverse/forward primer set (10 μM), 1 μL of 50× reverse transcriptase, and 12.8 μL of nuclease‐free water. To each reaction tube, 1 μL (10 ng) of plasmid control or 1 μL of purified plant DNA/RNA or 1–2 μL of direct crude plant extract was added followed by 2.5 μL of 280 mM magnesium acetate; the tube was mixed by pipetting and brief centrifugation. The tubes were immediately incubated at 39°C for 4 min, then it was rapidly inverted and mixed 6–10 times, followed by incubation at 39°C for 36 min. The RPA reactions included a negative control of no template control (NTC) and healthy plant control in each reaction. The RPA‐amplified products were further purified using a QIAquick PCR purification kit (Qiagen) then treated with 6× loading dye and SDS Sol (Thermo Scientific), and the purified RPA products were analysed by 1.5% agarose gel electrophoresis stained with SYBR Safe gel stain (Thermo Scientific), followed by imaging using the Alpha Imager HP system (Protein Simple).

**TABLE 1:** Primers targeting viral coat protein genes.

Virus	Primer number	Name	Sequence (5′–3′)	*T*_m_ (°C)	GC (%)	Amplicon (bp)
CCYV	MP69	CCYV‐CP‐F	GTTGTTTATTCAAATAGGGCAGATGTGATG	65	36.7	350
	MP70	CCYV‐CP‐R	TTAAGAATTTCAATATCTGACTCGTGTTTCC	64	32.3	
CuLCrV	MP71	CuLCrV‐CP‐F	CCGTCGACCGTATAGTTCTCCTATGGATTTC	68	48.4	273
	MP72	CuLCrV‐CP‐R	CATGCCATATACAATAACAAAGCGTTCTCAGTATG	68	37.1	
CYSDV	MP81F	CYSDV‐CP‐F	GCATCGGCTATATCAGTACAATACTTATCTTT	65	34.4	440
	MP82R	CYSDV‐CP‐R	GATCCATCTTTGGACTTCTCATCTTTCTTT	65	36.7	

Abbreviations: CCYV, cucurbit chlorotic yellows virus; CuLCrV, cucurbit leaf crumple virus; CYSDV, cucurbit yellow stunting disorder virus.

The duplex and multiplex RT‐RPA reactions were evaluated using mixtures of plasmid positive controls. The RPA reactions were standardized in four stages using the TwistDx RPA kit. In the first stage of standardization, the RPA reaction was carried out at three different temperatures: 38, 39, and 40°C. Then, amplicons were run in agarose gel to find the ideal temperature. After selection of the temperature, the mixed primer concentrations were optimized in different ratios: standard ratio (10 μM each primer of CCYV, CuLCrV and CYSDV), low and high ratios (CCYV 9 μM, CuLCrV 9 μM and CYSDV 12 μM; CCYV 12 μM, CuLCrV 9 μM and CYSDV 9 μM). The third stage of optimization was analysis of the primers at different concentrations (0.24, 0.28, 0.32, 0.36, 0.42 μM) in the multiplex RPA reaction. The final stage of standardization of RPA reaction used analysis at incubation times of 20, 30 and 40 min. The final and optimized multiplex RPA reaction was performed with the following reaction conditions: temperature 39°C, primer concentrations CCYV 9 μM, CuLCrV 9 μM and CYSDV 12 μM, primer mixture concentration 0.32 μM and incubation time 40 min.

The sensitivity of multiplex RPA assay was evaluated using a mixture of positive targets containing each CP plasmid at equal concentrations. The multiplex RPA master mix consisted of the following ingredients: 1.6 μL of each forward and reverse primer (added six primers at the following concentrations: CCYV 9 μM, CuLCrV 9 μM and CYSDV 12 μM; Table 1), 29.5 μL of 1× rehydration buffer and 13.8 μL of DNase‐free water. The master mix was mixed well (46.5 μL) and aliquoted into each tube containing the RPA pellet and then the RPA pellet was dissolved by mixing by rapid inversion. Thereafter, 1 μL of plasmid mixture (10^0^–10^5^ copies) and 2.5 μL of 280 mM magnesium acetate were added to the cap of the tube. The RPA reaction was mixed by inverting and repeating the process 6–10 times, with brief centrifugation before incubation. Each multiplex RPA reaction was conducted to detect the copy number using the purified plasmid quantified by a NanoDrop spectrophotometer. The multiplex RPA reaction was performed to detect a single copy using 10^0^–10^5^ serially diluted and equally mixed plasmid targets of CuLCrV, CYSDV and CCYV. The presence of tomato leaf curl New Delhi virus (ToLcNDV), CCYV, PRSV‐W, WMV‐2 and SqVYV was confirmed in the watermelon, squash and whitefly samples by simple one‐step RT‐PCR. The samples containing ToLcNDV (begomovirus) and CCYV (crinivirus) were used as inclusive panel controls, and those containing PRSV‐W, WMV‐2 (potyvirus) and SqVYV (ilarvirus) were used as exclusive panel controls for validation of the specificity of the multiplex RPA mixed primers.

The multiplex RT‐RPA master mix consisting of all ingredients as stated above and including Superscript Reverse Transcriptase IV (Thermo Scientific) was used to detect RNA viruses simultaneously in a single multiplex RPA reaction. Additionally, 1 μL of DNA/RNA/plant crude extracts and 2.5 μL of 280 mM magnesium acetate were added to the cap of the final reaction tube. The master mix preparation was prepared by the above‐described protocol, including RT enzyme. The final master mix was aliquoted into a TwistDx pellet‐containing tube followed by adding crude sap extracts and magnesium acetate to the cap of the tube. The tube was carefully closed, and the RPA ingredients were mixed properly with an immediately quick spin down followed by incubation of the reaction tube at 39°C for 40 min on a heat block. This protocol was followed to purify the multiplex RPA products and detect the RPA products in the gel by UV visualization.

The simple one‐step RT‐PCR results demonstrated that watermelon samples were positive for two viruses (CuLCrV and CYSDV) and squash samples were positive for all three viruses (Figure S1). Two sets of primers were screened in the RPA basic reaction and one of them was selected according to primer dimer formation (Figure S2). The selected primers were validated in singleplex RPA using the template as total plant nucleic acid and plasmid positive controls. The results showed that the expected amplicons were observed in the plant RNA and plasmid positive controls: 273, 350 and 440 bp, respectively (Figure 1a,d). The optimization of RPA temperature for the best amplification results showed that RPA amplicon products were increased when the temperature was increased from 37 to 39°C (Figure S3).

**FIGURE 1:**
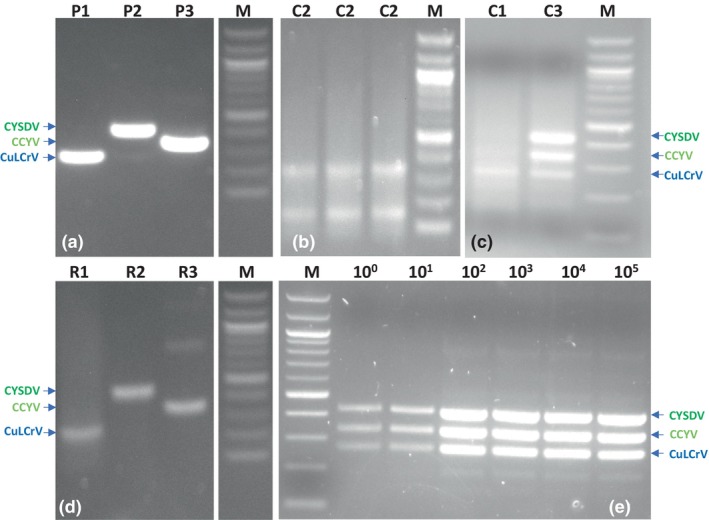
Sensitivity of the multiplex recombinase polymerase amplification (RPA) primers in singleplex, duplex and multiplex RPA reactions. Each primer set was tested in a singleplex RPA reaction using CuLCrV, CYSDV and CCYV plasmid mix (a) and symptomatic plant RNA (d). The standard primer concentration was used in singleplex RPA (c, lane c1). Optimized mixed primer concentration was used in duplex and multiplex RPA (b, lane c2; c, lane c3). The limit of detection was conducted using optimized multiplex RPA primers and 1–10^5^ copies of plasmids in a multiplex RPA reaction (e, lane: 10^0^–10^5^: 1, 10, 100, 1000, 10,000, and 100,000 copies). C, control mixed plasmids; CCYV, cucurbit chlorotic yellows virus; CuLCrV, cucurbit leaf crumple virus; CYSDV, cucurbit yellow stunting disorder virus; P, plasmid; R, plant RNA; M, marker (100 bp and 1 kb).

The single, double and triple primer concentrations were optimized according to the detection of single, double and triple viruses in a single RPA reaction. When the standard concentration of both primers (420 nM) was used in a singleplex RPA reaction, the results showed a prominent amplification band (Figure 1c, lane C1). The optimized primer concentrations of CuLCrV 9 μM and CYSDV 12 μM were used for duplex RPA reaction and resulted in the expected amplicons (Figure 1b, lane C2). The multiplex RPA primer concentrations were standardized using various primer concentrations and primer ratios in a single RPA reaction (Figures S3 and S4). The optimized concentrations of CCYV 9 μM, CuLCrV 9 μM and CYSDV 12 μM were used to obtain those amplicons in the multiplex PRA assay, and the band intensity was also equivalent to the band intensity observed in the singleplex RPA reaction (Figure 1c, lane C3).

The specificity test for the multiplex RPA primers was conducted using mixed plant RNA samples containing the viruses CuLCrV, CYSDV, CCYV, PRSV‐W, WMV‐2 and SqVYV, and the results were positive for CuLCrV, CYSDV and CCYV by single one‐step RT‐PCR and singleplex RPA (Figure S4). A sensitivity assay for the multiplex RPA primers was conducted to examine the level of detection using mixed plasmids by multiplex RPA. When 1 μL of the serially diluted plasmid (10^0^–10^6^ copies) was added to 10 μL of squash crude extracts, the results demonstrated that the primers amplified single‐copy targets in the multiplex RPA assay reaction (Figure 1e). The diluted plasmids used in the multiplex RT‐RPA assay were validated through serial dilution in the multiplex RT‐qPCR assay. Furthermore, pGEM‐T empty vector serially diluted 10^0^–10^5^‐fold was used as the negative control to assess the performance of the multiplex RPA primers. The results indicated that no band was observed when the empty vector was used, confirming the absence of amplification in the negative control (Figure S5). The second sensitivity assay for the RPA primers was conducted using both singleplex and duplex RPA reactions. The assay used 100 pg of total plant nucleic acid as the initial input. The results showed successful detection of both single and mixed viruses, while no amplification was observed in the RNA sample from a healthy plant (Figure 2a). When a third test was conducted using symptomatic and healthy squash and watermelon crude extracts using 1 μL of crude sap in multiplex RPA, the results showed the presences of amplicons, which were observed not only in one volume but also in two other volumes. No amplicon was observed in the healthy control with the different volumes tested (Figure 2b). The final sensitivity assay was conducted to detect the limit of amplification in the multiplex RPA reaction using different volumes of squash and watermelon crude extract that showed strong amplicons between 0.8 and 1.0 μL of sap (Figure 2c,d).

**FIGURE 2:**
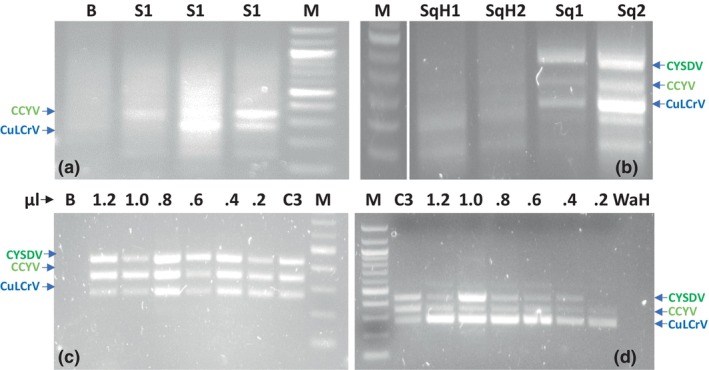
Sensitivity of the multiplex recombinase polymerase amplification (RPA) primers tested using 100 pg of symptomatic plant RNA and different volumes of symptomatic plant crude sap extracts. One hundred picograms of plant RNA was used in singleplex and duplex RPA reaction for primer sensitivity analysis (a). The multiplex RPA primers were tested using plant crude extracts of symptomatic and healthy samples in multiplex RPA (b). The crude extracts sensitivity assay was conducted with different volumes of squash and watermelon symptomatic samples (c: squash and d: watermelon 0.2–1.2 μL). B, buffer control; C3, control of mixed plasmids; M, marker; S1, sample 1 symptomatic RNA; Sq, squash symptomatic extracts; SqH, squash healthy; Wa, watermelon symptomatic extracts; WaH, watermelon healthy; CCYV, cucurbit chlorotic yellows virus; CuLCrV, cucurbit leaf crumple virus; CYSDV, cucurbit yellow stunting disorder virus.

The 15 samples, consisting of four asymptomatic watermelon samples, three symptomatic watermelon samples, and eight symptomatic squash samples, were initially tested using singleplex RT‐PCR. The results showed positive detection of double and mixed viruses, including CuLCrV, CYSDV, and CCYV, in the singleplex RT‐PCR assay (Table 2 and Figure S6). The same samples were tested in multiplex RPA assay using total nucleic acid and plant crude extracts. The expected results were observed, which had already been confirmed by singleplex RT‐PCR and singleplex RPA (Figure 3a,b RNA and Figure 3c,d crude extract).

**TABLE 2:** Testing of squash and watermelon samples for the presence of the CCYV, CuLCrV and CYSDV by reverse transcription (RT)‐PCR and multiplex RT‐recombinase polymerase amplification (RPA) assay.

Host	Sample	Symptom	Cultivar	Detection by single/multiplex RT‐RPA		
				CCYV	CYSDV	CuLCrV
Squash	201	+	Gentry	+/+	+/+	+/+
	202	+	Conqueror	+/+	+/+	+/+
	203	+	Respect	+/+	+/+	+/+
	204	+	Spineless Perfection	+/+	+/+	+/+
	205	+	Perfection	+/+	+/+	+/+
	206	+	Spineless Beauty	+/+	+/+	+/+
	207	+	Beauty	+/+	+/+	+/+
	208	+	Lioness	+/+	+/+	+/+
Watermelon	Sap 1	+	–	−/−	+/+	+/+
	Sap 2	+	–	−/−	+/+	+/+
	Sap 3	+	–	−/−	+/+	+/+
	Sap 4	−	–	−/−	−/−	+/+
	Sap 5	−	–	−/−	−/−	+/+
	Sap 6	−	–	+/+	+/+	+/+
	Sap 7	−	–	+/+	+/+	+/+

Abbreviations: CCYV, cucurbit chlorotic yellows virus; CuLCrV, cucurbit leaf crumple virus; CYSDV, cucurbit yellow stunting disorder virus.

**FIGURE 3:**
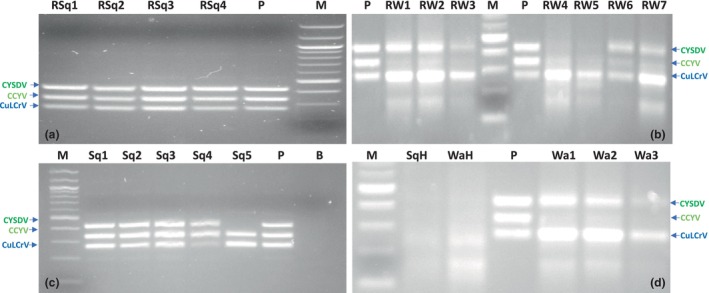
The simultaneous detection of CYSDV, CCYV and CuLCrV was performed using a multiplex reverse transcription‐recombinase polymerase amplification (RT‐RPA) assay. Total plant RNA samples, as well as crude extracts from symptomatic and healthy squash and watermelon plants, were used. The following labels were assigned to the different samples: squash and watermelon RNA (a, b); squash and watermelon plant crude extracts (c, d). P, plasmid control; RSq, squash symptomatic plant total RNA; RW, watermelon symptomatic plant total RNA; Sq, squash symptomatic plant crude extracts; SqH, squash healthy; Wa, watermelon symptomatic crude extracts; WaH, watermelon healthy.

The current study demonstrates the development of a simple and rapid isothermal multiplex RPA assay for the simultaneous detection of mixed DNA and RNA viruses (CuLCrV, CYSDV and CCYV) that are transmitted by whiteflies and affect many cucurbit crops. This rapid multiplex RPA assay has been created to detect single‐copy mixed DNA and RNA viruses from plant extracts using a single‐pellet RPA system. This study shows that detecting mixed viruses in the early infection stage and diagnosing mixed virus‐infected samples within a short time is achievable. These mixed viruses are an important threat to cucurbit production (Akad et al., 2008) and to the best of our knowledge a multiplex RPA diagnostic technique has not previously been reported for cucurbit‐infecting viruses as well as for other plant viruses. The assay is specific and sensitive, from as little as single‐copy detection when plant extracts are used as a template.

Many reports are available for plant pathogen detection through molecular and serological techniques such as PCR, RT‐PCR, RT‐qPCR, multiplex RT‐PCR, multiplex RT‐qPCR, ELISA and lateral flow test (Hosseini et al., 2018; Idris et al., 2008; Ling et al., 2010). Molecular techniques are specific and reliable, and diagnostic methods are expensive and require a modern laboratory and technical expertise that can detect nucleic acid to some extent and cannot detect fewer than 100 copies. On the contrary, serological‐based techniques are a cheaper but time‐consuming process but can bind nonspecific targets (Hosseini et al., 2018). The RPA technique avoids nucleic acid extraction and thus allows users to perform a multiplex diagnostic assay with ease (Silva et al., 2018). The singleplex RPA‐based detection assay has been previously reported for some common human, animal and plant pathogens (Castellanos‐Gonzalez et al., 2018; Wang et al., 2017; Wu et al., 2016). Duplex and multiplex RPA assays have also been developed against some human, animal and plant pathogens (Babu et al., 2017; Kalischuk et al., 2020; Lei et al., 2020; Wang et al., 2017), but have faced challenges in consistency of detection. The cucurbit‐infecting RNA and DNA viruses of CGMMV, CYSDV, CuLCrV and CMV all were previously reported to be detectable through a singleplex RT‐RPA assay but also not detected from single‐copy virus level (Jiao et al., 2019; Kalischuk et al., 2020, 2021; Srivastava et al., 2019). Thus, the developed isothermal‐based multiplex RPA detection assay for simultaneous detection of mixed DNA and RNA plant viruses by a single RPA pellet system provides new benefits.

In our study, we optimized the detection techniques to achieve high sensitivity in detecting the virus copies in both symptomatic and asymptomatic plant leaves (early infection stage). The developed multiplex RPA system detected multiple (mixed) targets of CuLCrV, CYSDV and CCYV from plasmid controls down to a single copy. The assay detected mixed viruses in direct plant crude extracts with volumes as low as 0.2 μL of sap when using the standardized primer mixer and temperature conditions. Compared to this study, previously developed singleplex RPA assays detected 100 fg for CYSDV, 10 fg for CuLCrV, 130 pg for CGMMV and 3 pg for CMV (Jiao et al., 2019; Kalischuk et al., 2020, 2022; Srivastava et al., 2019).

In summary, the multiplex RT‐RPA detection tool developed is specific, sensitive and highly efficient in detecting multiple virus targets in a single‐pellet RPA reaction tube. The developed multiplex RT‐RPA assay for simultaneous detection of the cucurbit‐infecting viruses CYSDV, CuLCrV and CCYV can be run within 80 min with up to eight samples able to be processed at a time. This is the first report of simultaneous detection of mixed plant DNA and RNA viruses by multiplex RT‐RPA assay. This multiplex RT‐RPA assay will be valuable for laboratories to detect viruses from a number of symptomatic and asymptomatic samples quickly and diagnose these viruses in the early infection period, which will greatly facilitate surveillance and minimize cost and time. Further research is required to evaluate the efficacy of this diagnostic method in early detection and asymptomatic tissue. Additional studies should be conducted to assess the sensitivity and specificity of the assay in detecting the target viruses in these specific conditions. This will help to determine the diagnostic's potential for early detection and identification of infections in asymptomatic plant tissues.

## CONFLICT OF INTEREST STATEMENT

We declare that no conflict of interest exists in the content of the manuscript.

## Supporting information


**Figure S1.** CCYV, CuLCrV and CYSDV viruses were detected in squash and watermelon leaf samples by one‐step reverse transcription‐PCR using coat protein gene‐specific primers. (a) CYSDV in watermelon lanes 1–4 and CuLCrV in watermelon lanes 5–8, (b) CuLCrV in squash lanes 9–12, (c) CYSDV in squash lanes 13–16, (d) CCYV in squash lanes 17–20. M, 1 kb marker.

**Figure S2.** Screening of CCYV, CuLCrV and CYSDV recombinase polymerase amplification (RPA) primers in basic reverse transcription‐RPA reaction.

**Figure S3.** Optimization of reverse transcription‐recombinase polymerase amplification (RT‐RPA) temperature for excellent amplification. The RPA primers were tested at two different temperatures using positive controls in the reaction: (a) 37°C and (b) 39°C. Lanes 1, 6, 11, CuLCrV; lanes 2, 7, 13, CCYV; lanes 3, 8, 12, CYSDV; M, 100 bp DNA marker.

**Figure S4.** Testing of recombinase polymerase amplification (RPA) primer specificity using inclusion and exclusion control of CCYV, CYSDV, CuLCrV, SqVYV, WMV and PRSV in one‐step reverse transcription (RT)‐PCR and singleplex RT‐RPA assay. (a, b) RPA primers were detected only in CYSDV (lanes 1–5), CCYV (lanes 6–10) and CuLCrV (lanes 11–15) from the squash and watermelon samples by RT‐PCR. (c) RPA primers were detected in CuLCrV (lane 16), CYSDV (lane 17) and CCYV (lane 18) in squash samples by RT‐RPA. M, 1 kb and 100 bp ladder.

**Figure S5.** The assessment of multiplex recombinase polymerase amplification (RPA) primers included testing the serially diluted pGEM‐T empty vector as a negative control.

**Figure S6.** The symptomatic squash and asymptomatic watermelon samples were used for this work.

## Data Availability

The data that support the findings of this study are available from the corresponding author upon reasonable request.
