# Supplementary material for: Development of a multiplex RT‐RPA assay for simultaneous detection of three viruses in cucurbits
Source: Mol Plant Pathol. 2023 Jul 18;24(11):1443–50. doi: 10.1111/mpp.13380 (PMC10576173; doi:10.1111/mpp.13380)
Supplement: Supplementary file 1 — Figure S1. CCYV, CuLCrV and CYSDV viruses were detected in squash and watermelon leaf samples by one‐step reverse transcription‐PCR using coat protein gene‐specific primers. (a) CYSDV in watermelon lanes 1–4 and CuLCrV in watermelon lanes 5–8, (b) CuLCrV in squash lanes 9–12, (c) CYSDV in squash lanes 13–16, (d) CCYV in squash lanes 17–20. M, 1 kb marker. [file MPP-24-1443-s002.docx]

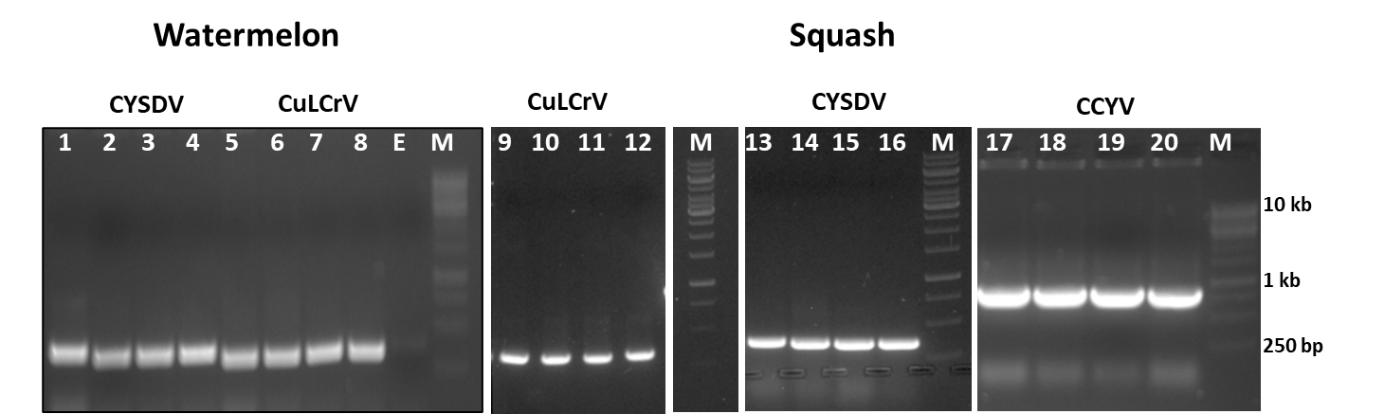


(a) (b) (c) (d)

**Sup Fig 1. CCYV, CuLCrV, and CYSDV viruses were detected in squash and watermelon leaf samples by one-step RT-PCR using CP gene-specific primers.** (a) CYSDV in watermelon lane:1-4 and CuLCrV in watermelon Lane 5-8, (b) CuLCrV in squash lane 9-12, (c) CYSDV in squash lane 13-16, (d) CCYV in squash Lane 17-20. M: 1 kb Marker.
