# Supplementary figures and images for: Development of a multiplex RT‐RPA assay for simultaneous detection of three viruses in cucurbits
Source: Mol Plant Pathol. 2023 Jul 18;24(11):1443–50. doi: 10.1111/mpp.13380 (PMC10576173; doi:10.1111/mpp.13380)

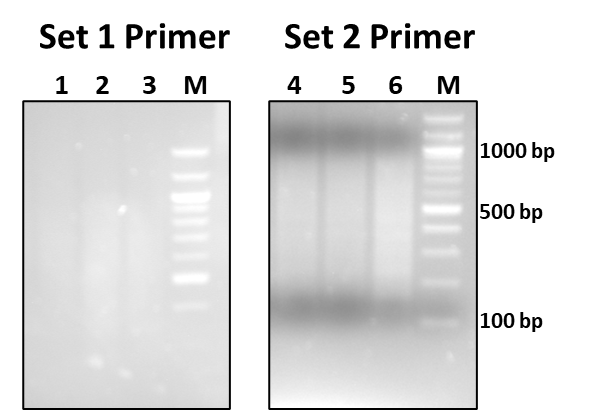


**Sup Fig 2. Screening of CCYV, CuLCrV, and CYSDV RPA primers in basic RT-RPA rection.**

Supplement: Supplementary file 2 — Figure S2. Screening of CCYV, CuLCrV and CYSDV recombinase polymerase amplification (RPA) primers in basic reverse transcription‐RPA reaction. [file MPP-24-1443-s004.docx]
