# Supplementary material for: Development of a multiplex RT‐RPA assay for simultaneous detection of three viruses in cucurbits
Source: Mol Plant Pathol. 2023 Jul 18;24(11):1443–50. doi: 10.1111/mpp.13380 (PMC10576173; doi:10.1111/mpp.13380)
Supplement: Supplementary file 3 — Figure S3. Optimization of reverse transcription‐recombinase polymerase amplification (RT‐RPA) temperature for excellent amplification. The RPA primers were tested at two different temperatures using positive controls in the reaction: (a) 37°C and (b) 39°C. Lanes 1, 6, 11, CuLCrV; lanes 2, 7, 13, CCYV; lanes 3, 8, 12, CYSDV; M, 100 bp DNA marker. [file MPP-24-1443-s005.docx]

**
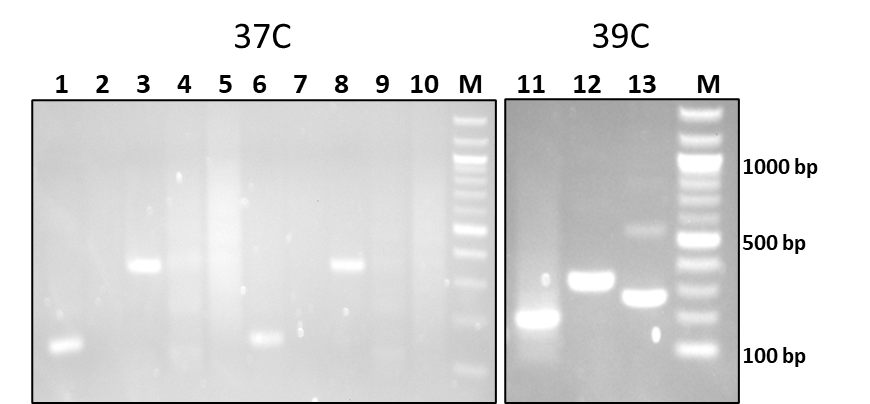
**

(a) (b)

**Sup Fig. 3 Optimization of RT-RPA temperature for excellent amplification**. The RPA primers were tested at two different temperatures using positive controls in the reaction (a) 37C and (b) 39C. Lane: 1,6, 11- CuLCrV; 2,7,13-CCYV; 3, 8, 12-CYSDV. M: 100 bp DNA Marker.
