# Supplementary material for: Development of a multiplex RT‐RPA assay for simultaneous detection of three viruses in cucurbits
Source: Mol Plant Pathol. 2023 Jul 18;24(11):1443–50. doi: 10.1111/mpp.13380 (PMC10576173; doi:10.1111/mpp.13380)
Supplement: Supplementary file 4 — Figure S4. Testing of recombinase polymerase amplification (RPA) primer specificity using inclusion and exclusion control of CCYV, CYSDV, CuLCrV, SqVYV, WMV and PRSV in one‐step reverse transcription (RT)‐PCR and singleplex RT‐RPA assay. (a, b) RPA primers were detected only in CYSDV (lanes 1–5), CCYV (lanes 6–10) and CuLCrV (lanes 11–15) from the squash and watermelon samples by RT‐PCR. (c) RPA primers were detected in CuLCrV (lane 16), CYSDV (lane 17) and CCYV (lane 18) in squash samples by RT‐RPA. M, 1 kb and 100 bp ladder. [file MPP-24-1443-s006.docx]

**
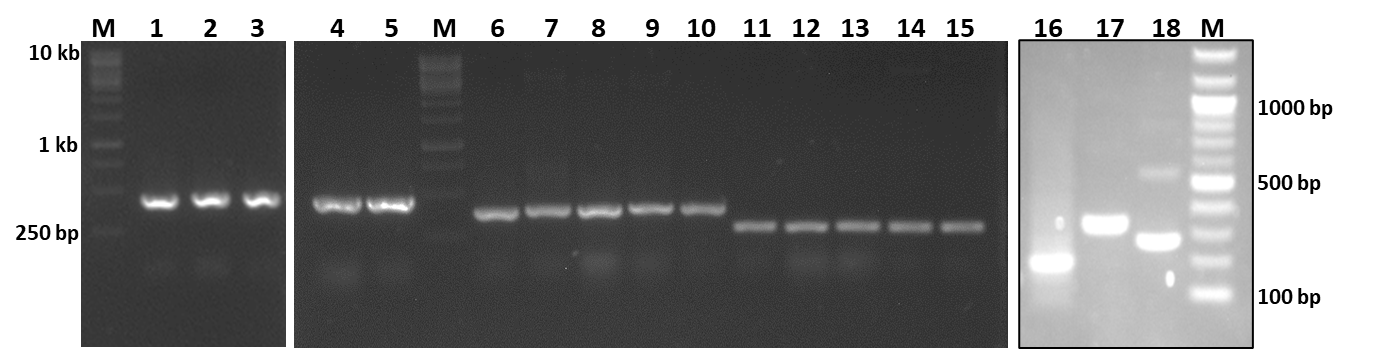
**

(a) (b) (c)

**Sup Fig4. Testing of RPA primers specificity using inclusion and exclusion control of CCYV, CYSDV, CuLCrV, SqVYV, WMV, and PRSV in one-step RT-PCR and singleplex RT-RPA assay.** (a,b) RPA primers were detected only in CYSDV (lanes 1-5), CCYV (lanes 6-10), and CuLCrV (Lane 11-15) from the squash and watermelon samples by RT-PCR; (c) RPA primers were detected in CuLCrV (lane 16), CYSDV (Lane 17) and CCYV (lane 18) in squash sample by RT-RPA. M: 1 kb and 100 bp ladder.
