# Supplementary material for: Development of a multiplex RT‐RPA assay for simultaneous detection of three viruses in cucurbits
Source: Mol Plant Pathol. 2023 Jul 18;24(11):1443–50. doi: 10.1111/mpp.13380 (PMC10576173; doi:10.1111/mpp.13380)
Supplement: Supplementary file 5 — Figure S5. The assessment of multiplex recombinase polymerase amplification (RPA) primers included testing the serially diluted pGEM‐T empty vector as a negative control. [file MPP-24-1443-s001.docx]

**Sup Fig. 5 The assessment of multiplex RPA primers included testing the serially diluted pGEMT empty vector as a negative control.**
