# Supplementary material for: Development of a multiplex RT‐RPA assay for simultaneous detection of three viruses in cucurbits
Source: Mol Plant Pathol. 2023 Jul 18;24(11):1443–50. doi: 10.1111/mpp.13380 (PMC10576173; doi:10.1111/mpp.13380)
Supplement: Supplementary file 6 — Figure S6. The symptomatic squash and asymptomatic watermelon samples were used for this work. [file MPP-24-1443-s003.docx]

**Sup Fig 6.** **The symptomatic squash and asymptomatic watermelon samples were used for this work**.
